# Taxonomic Status of *Diplectanum robustitubum* Wu & Li, 2003 (Monogenoidea: Diplectanidae) from the Purple-Spotted Bigeye *Priacanthus tayenus* (Priacanthidae) and the Description of *Platycephalotrema parile* n. sp. (Monogenoidea: Dactylogyridae) from the Bartail Flathead *Platycephalus indicus* (Platycephalidae), in the Arabian Gulf

**DOI:** 10.1007/s11686-024-00849-4

**Published:** 2024-05-25

**Authors:** Delane C. Kritsky, Ali Adnan Al-Darwesh, Atheer H. Ali

**Affiliations:** 1 School of Health Professions, Idaho State University, Campus, Box 8090, Pocatello, ID 83209 USA; 2 Department of Pathology and Poultry Diseases, College of Veterinary Medicine, University of Kufa, Kufa, Iraq; 3 Department of Fisheries and Marine Resources, College of Agriculture, Basrah University, Basrah, Iraq

**Keywords:** Arabian Gulf, Dactylogyridae, Diplectanidae, *Haliotrema swatowense*, Iraq, Kuwait, Monogenoidea, *Oliveriplectanum robustitubum* n. comb., *Platycephalotrema indicus* n. comb., *Platycephalotrema parile* n. sp., *Platycephalus indicus*, *Priacanthus tayenus*

## Abstract

**Abstract:**

Purple-spotted bigeyes *Priacanthus tayenus* Richardson (Priacanthidae) and bartail flathead *Platycephalus indicus* (Linnaeus) (Platycephalidae) were collected from the Arabian Gulf and examined for species of Monogenoidea (Polyonchoinea) from February to December 2020. *Diplectanum robustitubum* Wu & Li, 2003 and an undescribed species of *Platycephalotrema* Kritsky & Nitta, 2019 were recovered from the gill lamellae of these hosts, respectively. *Diplectanum robustitubum* from Iraq was redescribed and transferred to *Oliveriplectanum* Domingues & Boeger, 2008 (Diplectanidae) as *Oliveriplectanum robustitubum* (Wu & Li, 2003) n. comb. *Platycephalotrema parile* n. sp. (Dactylogyridae) from Iraq and Kuwait was described and differentiated from the similar species, *Haliotrema indicum* Tripathi, 1959, *Platycephalotrema ogawai* Kritsky & Nitta, 2019, and *Platycephalotrema platycephali* (Yin & Sproston, 1948) Kritsky & Nitta, 2019, based primarily on the comparative morphologies of the vaginal sclerites. *Haliotrema indicum* was transferred to *Platycephalotrema* as *Platycephalotrema indicum* (Tripathi, 1959) n. comb. and *Haliotrema swatowense* Yao, Wang, Xia, & Chen, 1998 was considered a junior subjective synonym of *P. indicum*. The finding of *O. robustitubum* in the Arabian Gulf represents a new geographic record for the species.

**Background:**

The present paper represents the third installment concerning the monogenoids collected during surveys to explore their diversity on the marine and freshwater fishes of Iraq. Previous installments on the monogenoids emanating from the surveys included the dactylogyrid and gyrodactylid species parasitizing mugilid fishes.

**Purpose:**

The purpose of this paper is to further document the diversity of monogenoids infecting the fishes of Iraq.

**Methods:**

Marine fishes were necropsied for parasites, and standard procedures for collecting, mounting, drawing, and measuring of monogenoids were employed.

**Results:**

*Oliveriplectanum robustitubum* n. comb. (Diplectanidae) and *Platycephalotrema parile* n. sp. (Dactylogyridae) were collected. The occurrence of *O. robustitubum* in the Arabian Gulf represented a new locality record for the species.

**Conclusion:**

The recorded presence of *O. robustitubum* and *P. parile* n. sp. suggests that the diversity of monogenoids in Iraq is under estimated in the literature.

## Introduction

The diversity of monogenoids infecting the marine fishes of Iraq is poorly documented and continues to receive minimal attention by investigators. Ali et al. [4] reported that 322 species of marine fishes had been recorded in the territorial marine waters, marshes, and the lower reaches of the Euphrates and Tigris Rivers of Iraq during the period of 1874 to mid-2018, whereas Mhaisen et al. [17] listed only 21 fully identified valid species of Monogenoidea (including one species considered *species inquirenda*) having been recorded from these fishes through 2017. Since the list of Mhaisen et al. [17], three species of Diplectanidae infecting marine fishes of Iraq have been added: *Lamellodiscus indicus * Tripathi, 1959 from the haffara seabream *Rhabdosargus haffara* (Forsskål) and goldlined seabream *Rhabdosargus sarba* (Forsskål) (both Sparidae) [2], *Protolamellodiscus senilobatus* Kritsky, Jiménez-Ruiz and Sey, 2000 from the king soldierbream *Argyrops spinifer* (Forsskål) (Sparidae) [2], and *Calydiscoides difficilis* (Yamaguti, 1953) Young, 1969 from the pink ear emperor *Lethrinus lentjan* (Lacepède), the spangled emperor *Lethrinus nebulosus* (Forsskål) (both Lethrinidae), and the areolate grouper *Epinephelus areolatus* (Forsskål) (Epinephelidae) [3]. The latter three species were included in the 12 dactylogyrid and 16 diplectanid species recorded and/or described from Iraqi marine fishes in an unpublished thesis by Al-Darwesh [1].

In the present paper, the taxonomic status of the diplectanid *Diplectanum robustitubum*  Wu & Li, 2003 and that of the dactylogyrid *Platycephalotrema parile* n. sp. are evaluated. Both helminths occur on the gill lamellae of marine fishes of Iraq: *D. robustitubum* on the purple-spotted bigeye *Priacanthus tayenus* Richardson (Priacanthidae) and *Pt. parile* n. sp. on the bartail hardhead *Platycephalus indicus* (Linnaeus) (Platycephalidae). The respective hosts occur naturally throughout much of the Indo-West Pacific Ocean, where both are commercially important. Although less so in other regions, bigeyes comprise a significant component of the trawl fishery of southeast Asia, particularly in the areas if the Andaman Sea and the southern region of the South China Sea [23]. Within the middle eastern region of Asia, the bartail flathead is the most common flathead appearing in markets [9], where it is sold as a very palatable food fish [5]; the flathead is also a component of Chinese medicine [24].

## Materials and Methods

Specimens of the purple-spotted bigeye and the bartail hardhead were collected off the southern marine coast of Iraq near the mouth of the Shatt Al-Arab River (29°53′–29°85′N, 48°13'–48°40′E) by fishermen using trawl nets during February through December, 2020, where salinity levels varied between 24.5–32.5 PSU. Fish hosts were identified using Carpenter et al. [9],the higher classification for the fishes was that presented by Betancur-R et al. [6],and common and scientific names of fishes were verified using Froese & Pauly [12] and Fricke et al. [11], respectively. The fishes were transported in an icebox filled with crushed ice to the laboratory located in Basrah, Iraq, and examined for monogenoids within 48 h of capture. Gill baskets were removed and place in vials containing a hot (~ 60 C) 5% formalin (2% formaldehyde) solution for relaxation and fixation of monogenoids. The vials were labeled and then shaken to ensure suitable fixation and removal of helminths from the gill tissues. Helminths were subsequently picked from the gills or usually from the sediment using a fine probe, placed in small labeled vials, and shipped to Idaho State University for study. Methods for preparation, illustration, and measurement of monogenoidean specimens were those of Kritsky [13], see Sey & Nahhas [22] for methods for collection and preservation of specimens from Kuwait. Illustrations were prepared using a microprojector or a camera lucida mounted on a compound phase-contrast microscope. Measurements, all in micrometers, represented straight-line distances between extreme points and were expressed as the mean followed by the range and number (n) of structures measured in parentheses; body length included that of the haptor. Numbering (distribution) of haptoral hooks followed the convention proposed by Mizelle ([18], see Mizelle & Price [19]; direction of the coil of the shaft of the MCO, clockwise vs. counterclockwise, was determined using the method proposed by Kritsky et al. [15]. Definitions of ecological terms were those of Bush et al. [7], except that prevalence and intensity were reported as “minimum prevalence” and “minimum intensity” sensu Kritsky et al. [14]. Voucher specimens of the helminths were deposited in the Invertebrate Zoology Collection, National Museum of Natural History, Smithsonian Institution, Washington, D. C. (USNM) and the University of Nebraska State Museum, Harold W. Manter Laboratory, Lincoln, Nebraska (HWML), as indicated in the following species accounts. For comparative purposes, the holotypes of *Diplectanum curvivagina*  Yamaguti, 1968 (USNM 1359364), *Diplectanum opakapaka* Yamaguti, 1968 (USNM 1359368), and *Diplectanum priacanthi* Yamaguti, 1968 (USNM 1359369) and paratypes of *Platycephalotrema ogawai* Kritsky & Nitta, 2019 (HWML 216008) were examined. Type specimens of *D. robustitubum* and *P. indicum* were not available for study.

In the various sections of this paper, the following abbreviations for host and parasite genera beginning with the letter “P” are utilized: *Pr*. for *Priacanthus*, *Pl*. for *Platycephalus*, and *Pt*. for *Platycephalotrema*. These abbreviations were only used after the respective generic and appended specific names were first presented within a given passage or paragraph.

## Results and discussion

The gill lamellae of 71 *Priacanthus tayenus* and 295 *Platycephalus indicus* collected from the Arabian Gulf off Iraq during January to December 2020 were examined for monogenoids. Each host species was infected with one species of Monogenoidea: *Diplectanum robustitubum* Wu & Li, 2001 (Diplectanidae) on *Pr. tayenus* and an undescribed species of *Platycephalotrema* (Dactylogyridae) on *Pl. indicus*. A minimum prevalence of 19.7% (14 of 71 fishes examined) and a mean minimum intensity of 13 (8–20) parasites per host were observed for *D. robustitubum*; the minimum prevalence for *Platycephalotrema* sp. was 80.3% (237 of 295), the minimum intensity was not recorded for this species. The taxonomic status of each of the two helminth species along with their respective descriptions are provided below.

Class Monogenoidea Bychowsky, 1937.

Subclass Polyonchoinea Bychowsky, 1937.

Order Dactylogyridea Bychowsky, 1937.

Diplectanidae Monticelli, 1903.

*Oliveriplectanum* Domingues & Boeger, 2008 

**Figs. 1−7:**
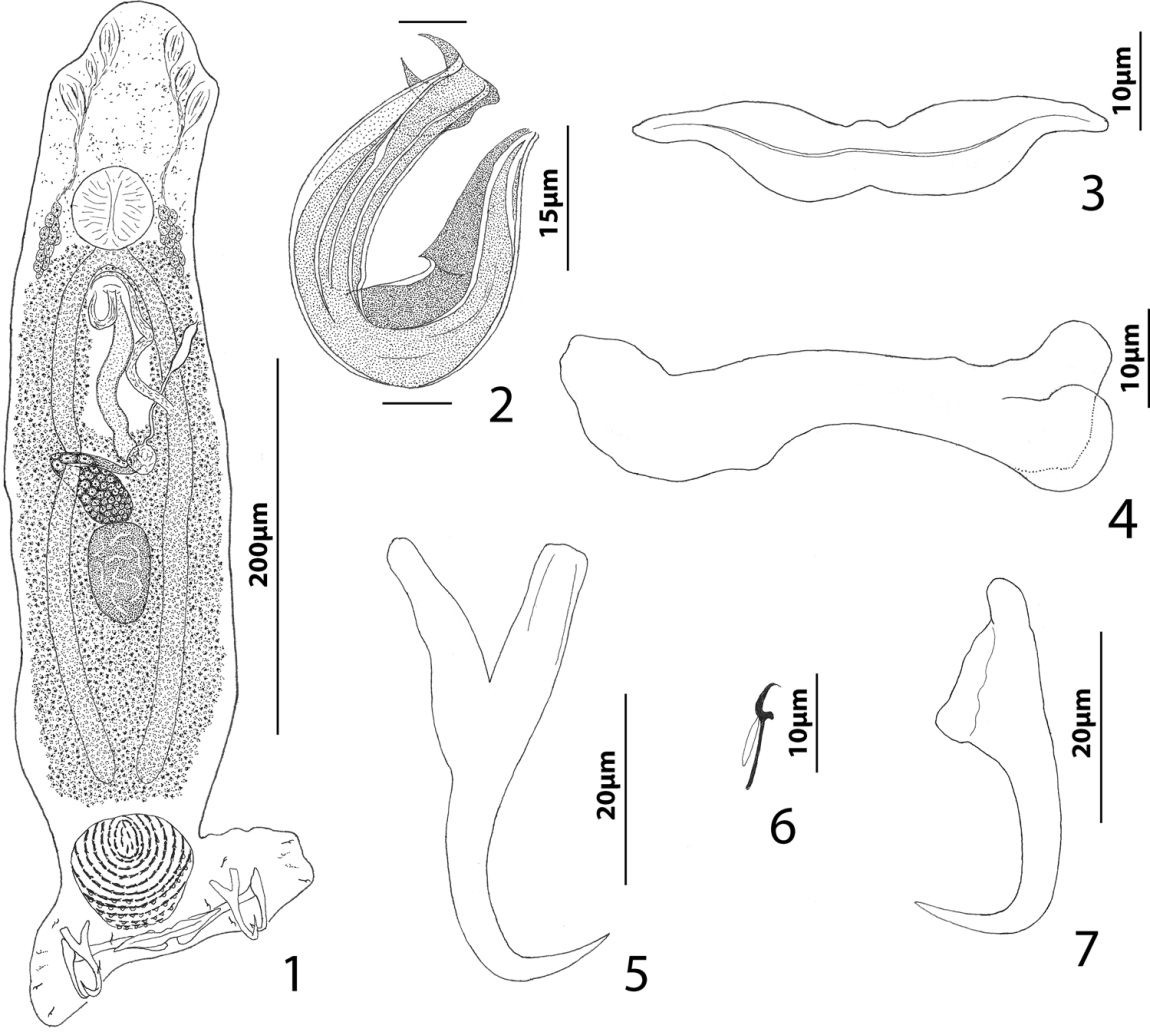
*Oliveriplectanum robustitubum* (Wu & Li, 2003) n. comb. infecting *Priacanthus tayenus*. 1―whole mount (composite, ventral view), 2―male copulatory organ (ventral view), 3―Ventral bar, 4―dextral dorsal bar (ventral view), 5―ventral anchor, 6―hook, 7―dorsal anchor. Parallel lines on Fig. 2 indicate the limits of the dimension measured.

*Oliveriplectanum robustitubum  *(Wu & Li, 2003)  n. comb. (Figs. 1–7). Syn. *Diplectanum robustitubum* Wu & Li, 2003.

*Type host*: Purple-spotted bigeye *Priacanthus tayenus* Richardson, Priacanthidae.

*Type locality*: Sanya, Hainan Province (18°18′N, 109°27′E) and Huizhou, Guangdong Province, China.

*Current record*: *Pr. tayenus*: Arabian Gulf off southern Iraq (29°50–29°51′N, 48°28′–48°38′E), February 2020–December 2020.

*Previous record*: There have been no other records of the species since its original description by Wu & Li [26].

*Infection site*: Gill lamellae.

*Specimens studied*: 27 voucher specimens, USNM 1707482–1707497, HWML 217614.

*Museum specimens examined*: Holotype, *Diplectanum curvivagina* Yamaguti, 1968 , USNM 1359364, holotype, *Diplectanum opakapaka* Yamaguti, 1968, USNM 1359368, holotype, *Diplectanum priacanthi* Yamaguti, 1968, USNM 1359369.

*Redescription*: Body proper fusiform, slightly flattened dorsoventrally; greatest width (excluding haptor) at level of gonads. Cephalic region with rounded medial and two moderately developed bilateral cephalic lobes; three bilateral pairs of head organs; cephalic glands composed of two bilateral groups of glandular cells lying posterolateral to pharynx. Tegument smooth. Eyespots absent; chromatic granules small, ovate, numerous, scattered throughout cephalic region, often forming small clumps anterior to pharynx. Mouth midventral at level of anterior margin of pharynx; pharynx subspherical; esophagus short to non-existent; intestinal ceca two, lacking diverticula, terminating blindly in posterior trunk. Peduncle short, tapered posteriorly; haptor with dorsal and ventral squamodiscs and two bilateral lobes containing hook pairs 2–4, 6, and 7. Dorsal and ventral squamodiscs usually with 11 rows of rodlets each (varying from 9 to 12); deep (anterior-most) one to two rows forming closed circles; rodlets of anterior seven rows of squamodisc dumb-bell shaped, those of rows 8–11 with posterior shield-like processes. Ventral anchor with deep and superficial roots of base subequal in length, gently arced shaft, recurved point. Dorsal anchor with subtriangular base, slightly arced to straight shaft, recurved point. Ventral bar with medial constriction, tapered ends, longitudinal ventral groove. Paired dorsal bars; each with bilobed medial end and anteriorly directed lateral end. Hook with protruding terminally blunt thumb, slender shank comprised of single subunit; filamentous hook loop about 3/4 shank length. Common genital pore ventral near level of intestinal bifurcation. MCO a U- or C-shaped tube with internal (nested sensu [10]) tubular canal and distal thorn-like processes. Accessory piece absent. Gonads tandem, testis postgermarial. Testis ovate. Proximal portion of vas deferens not observed; seminal vesicle inconspicuous, a slightly dilated segment of distal vas deferens, lying to left of body midline, looping anterior to MCO before forming posteriorly directed ejaculatory duct. Prostates, prostatic reservoir not observed. Germarium pyriform, dorsoventrally looping right intestinal cecum. Oötype, Mehlis’ glands not observed. Uterus dilated, extending from region anterior to germarium along body midline to common genital pore. Vaginal pore sinistroventral, submarginal; elongate vaginal vestibule giving rise to delicate vaginal canal. Seminal receptacle overlying anterior end of germarium and oviduct. Vitellarium dense, coextensive with intestinal ceca; transverse vitelline ducts at level of seminal receptacle. Egg not observed.

*Measurements* (corresponding measurements provided by Wu & Li [26] follow in brackets those obtained during the present study)**:** Body 437–624 (541; n = 15) [487–676 (575)] long; greatest width of trunk 86–142 (119; n = 15) [104–228 (127)]. Haptor 139–195 (162; n = 15) [169–197 (180)] wide. Ventral haptoral disc 47–62 (54; n = 13) long, 46–67 (57; n = 13) wide; dorsal haptoral disc 45–62 (53; n = 13) long, 46–64 (55; n = 13) wide. Ventral anchor 48–62 (51; n = 11) [42–49 (46)] long; dorsal anchor 41–45 (43; n = 12) [38–42 (40)] long. Ventral bar 52–65 (59; n = 10) [65–75 (73)] long; dorsal bar 60–71 (66; n = 12) [65–78 (72)] long. Hook 10–12 (11; n = 15) [8–10 (9.2)] long. Pharynx 36–48 (42; n = 14) long, 35–48 (39; n = 14) wide [39–47 (43) in diameter]. Testis 50–77 (63; n = 14) long, 29–58 (42; n = 14) wide. MCO 38–47 (43; n = 12) [34–46 (42)] long. Germarial bulb 27–42 (34; n = 14) wide.

### Remarks

Based on a cladistic analysis of morphological characters possessed by some members of the Diplectanidae, Domingues & Boeger [10] proposed *Oliveriplectanum* Dominques & Boeger, 2008 (Monogenoidea: Diplectanidae) for three species formerly assigned to *Diplectanum* Diesing, 1858: the type species *Oliveriplectanum priacanthi* (Yamaguti, 1968) Domingues & Boeger, 2008  from the glasseye *Heteropriacanthus cruentatus* (Lacepède), Priacanthidae, *Oliveriplectanum opakapaka* (Yamaguti, 1968) Domingues & Boeger, 2008  from the crimson jobfish *Pristipomoides filamentosus* (Valenciennes) and the rusty jobfish *Aphareus rutilans* Cuvier, both Lutjanidae; and *Oliveriplectanum curvivagina* (Yamaguti, 1968) Domingeus & Boeger, 2008 [ from the lavender jobfish *Pristipomoides sieboldii* (Bleeker) and goldflag jobfish *Pristipomoides auricilla* (Jordan, Evermann, & Tanaka), both Lutjanidae. Domingues & Boeger [10] were apparently unaware of the description of *Diplectanum robustitubum* by Wu & Li [26], as the present authors initially were, as indicated by the species being unlisted as a member of the Diplectanidae in their paper.

In addition to characters termed “traditional” by Dominques & Boeger [10], i.e., apparently characters not included in their analysis and apparently ubiquitous among diplectanid species, *Oliveriplectanum* was characterized by species having three features that they identified as plesiomorphies and autapomorphies: (1) the genital pore opening anterior to the MCO, (2) the anterior rows of rodlets of the squamodiscs being closed, and (3) the superficial root of the ventral anchor “developed” (i.e., the superficial and deep roots of the ventral anchor subequal in length). The latter three characters represented either autapomorphic reversals to the respective plesiomorphic states or parallel developments of features occurring in other ingroup taxa. Autapomorphic features unique to members of *Oliveriplectanum*, i.e., features not present in any other ingroup taxon in the analysis, were not identified. However, the absence of a unique autapomorphy for *Oliveridiplectanum* does not necessarily mean that the genus is invalid. Whereas a character state present in a common ancestor or other ingroup taxa may be impossible or difficult to differentiate from the respective feature found in species of *Oliveriplectanum*, these characters in the respective taxa are not homologs as each apparently has a unique evolutionary history. Thus, assuming that the hypothesis presented by Domingues & Boeger [10] actually represents the evolutionary history of the Diplectanidae, *Oliveriplectanum* is here accepted as valid.

The proposed transfer of *D. robustitubum* to *Oliveriplectanum*, as *Oliveriplectanum robustitubum* (Wu & Li, 2003)  n. comb., is based on the species having of all of the so-called plesiomorphic and autapomorphic characters defining the genus (see [10]). The species resembles *O. priacanthi and O. curvivagina* by having a U- or C-shaped MCO, an uncommon feature among members of the Diplectanidae including their remaining congener *O. opakapaka* that possesses a coiled MCO with several apparently counterclockwise rings. *Oliveriplectanum robustitubum*, with one to three rows of the squamodiscs forming closed circles, differs from *O. priacanthi* with apparently all rows of the squamodiscs being closed (see Fig. 93b in [30]) and from *O. curvivagina* with at least eight of ten rows closed in its squamodiscs (see Fig. 88b in [30]). The species also differs from *O. priacanthi* and *O. curvivagina* by having distal thorn-like projections on the MCO (absent in the latter two species). Finally, *O*. *robustitubum* and *O. priacanthi* lack eyespots, whereas two pairs are present in *O. curvivagina* and *O. opakapaka*.

Dactylogyridae Bychowsky, 1933.

*Platycephalotrema* Kritsky & Nitta, 2019. 

*Platycephalotrema parile* n. sp. (Figs. 8–16).

**Figs. 8−14:**
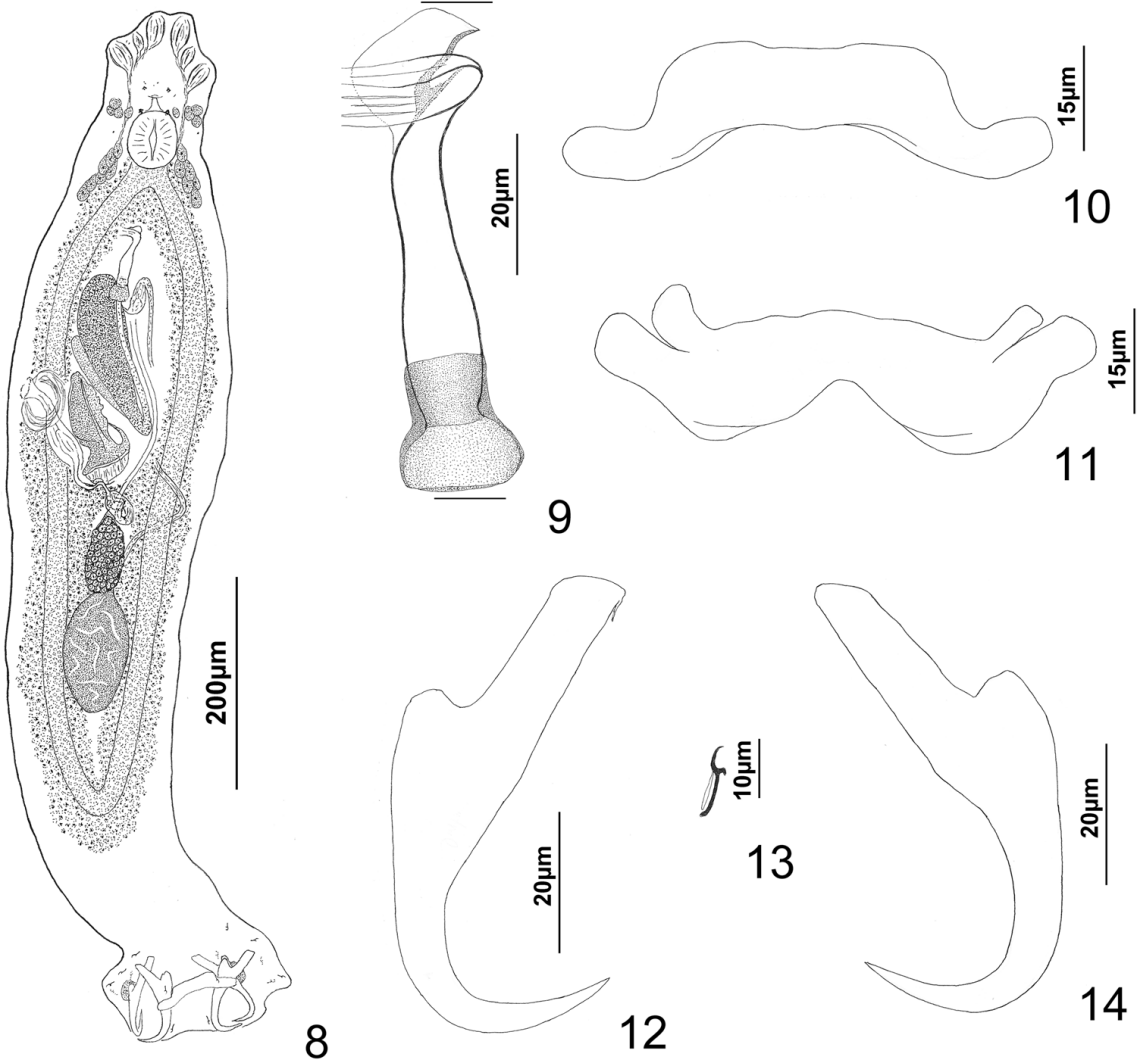
*Platycephalotrema parile* n. sp. infecting *Platycephalus indicus*. 8―whole mount (composite, ventral view), 9―male copulatory complex (ventral view), 10―ventral bar, 11―dorsal bar, 12―ventral anchor, 13―hook, 14―dorsal anchor. Parallel lines on Fig. 9 indicate the limits of the dimension measured.

**Figs. 15−17:**
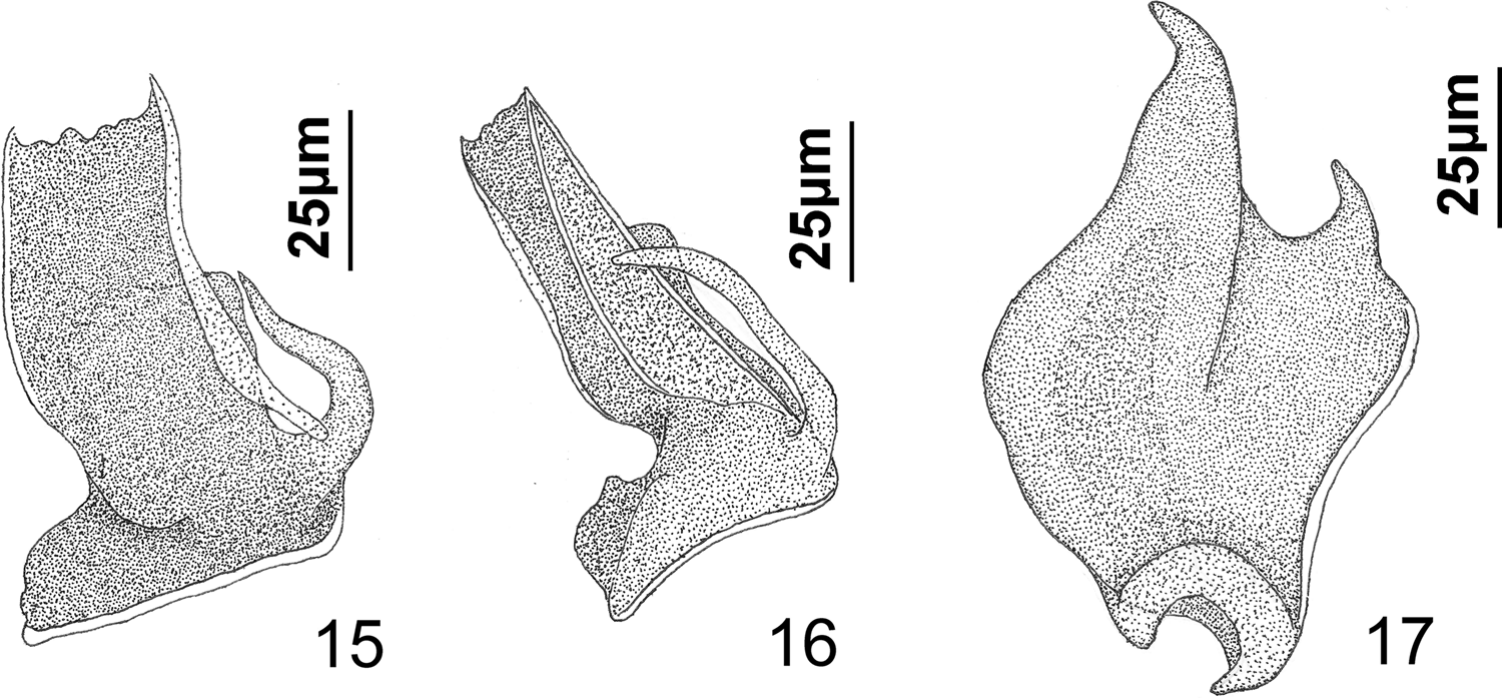
Ventral views of vaginal sclerites of *Platycephalotrema parile* n. sp. (Figs. 15, 16) and *Platycephalotrema ogawai* Kritsky & Nitta, 2019 (Fig. 17).

*Type host*: Bartail flathead *Platycephalus indicus* (Linnaeus), locally known as Wahara (وحره.), Platycephalidae.

*Type locality*: Arabian Gulf off southern Iraq (29°50–29°51′N, 48°28′–48°38′E), February 2020–December 2020.

*Other record*: *P. indicus*: Arabian Gulf off Kuwait, 15 June 1993, 21 December 1993.

*Infection site*: Gill lamellae.

*Specimens studied*: Holotype, USNM 1707420; 29 paratypes, USNM 1707421–1707436 HWML 217611; 12 voucher specimens (from Kuwait), USNM 1707437–1707448.

*Museum specimens examined*: 7 voucher specimens of *Platycephalotrema ogawai* Kritsky & Nitta, 2019  from the type host *Platycephalus* sp., locally known as “Yoshino-gochi” (ヨシノゴチ), HWML 216008.

*Etymology*: The specific name is from Latin (*parilis* = equal or like) and refers to the species resembling other species infecting the bartail flathead in the Indo-Pacific Ocean.

*Zoobank registration*: urn:lsid:zoobank.org:act:C8416F0A-5E17-46FE-835D-BAED16B345A9.

*Description*: Based on the specimens from Iraq. Greatest width of body proper usually near midlength of trunk. Cephalic region with well-developed medial and bilateral cephalic lobes. Eyespots four, apparently lacking lenses; individual eyespots occasionally dissociated; members of respective pairs equidistant, those of posterior pair larger than those of anterior pair. Accessory chromatic granules absent or few in cephalic region. Pharynx ovate; esophagus short to absent. Peduncle moderately long, slightly tapered posteriorly. Haptor subhexagonal, with lateral lobes containing hook pairs 3, 4, 6, 7; bilateral vesicles filled with granular material, lying lateral to bases of dorsal anchors. Ventral and dorsal anchors similar; each with elongate superficial root, short knob-like deep root, slightly arced to straight shaft, elongate point. Ventral bar with long spatulate ends directed laterally in haptor. Dorsal bar variable in width along its length, with unequal terminal limbs directed anterolaterally. Hook with uniform shank, conspicuous protruding blunt thumb; FH loop nearly shank length. MCO with inverted-bowl-shaped base, slightly tapering tubular shaft; distal end of shaft with clockwise terminal loop and weakly sclerotized flap. Testis ovate; proximal vas deferens dorsoventrally looping left intestinal cecum, distal vas deferens expanding to form fusiform seminal vesicle lying to left of MCO. Prostates not observed; two large prostatic reservoirs; anterior (ventral) reservoir usually containing two poorly differentiated zones of granules; posterior (dorsal) reservoir smaller than and often overlain by anterior reservoir. Germarium ovate; oviduct, oötype, Mehlis’ glands not observed; uterus delicate, diverted to left of large vaginal sclerite and prostatic reservoirs. Vaginal pore dextroventral, submarginal, surrounded by delicate sphincter-like muscle; vaginal ampulla lying posterolateral to vaginal sclerite and with comparatively thin wall; vaginal canal delicate, meandering from ampulla to small seminal receptacle. Vaginal sclerite appearing morphologically variable depending on degree of rotation within body, with anteriorly directed spine originating from base, planate distal flap having thickened margins (Fig. 15); anterior margin of flap often folded posteroventrally; fold, when present, appearing as a tapered spine (Fig. 16); base of vaginal sclerite resting on muscular pad. Seminal receptacle ovate, lying immediately anterior to germarium. Vitellarium dense, coextensive with intestinal ceca; transverse vitelline duct at level of seminal receptacle. Egg not observed.

*Measurements*: Body 768–1,180 (955; n = 15); greatest width of trunk 147–216 (180; n = 14). Haptor 138–195 (159; n = 14) wide. Ventral anchor 62–84 (75; n = 15) long; dorsal anchor 59–80 (72; n = 15) long. Ventral bar 64–78 (73; n = 13) long; dorsal bar 72–82 (76; n = 9) long. Hook 12–14 (13; n = 23) long. Pharynx 43–67 (55; n = 15) long, 40–67 (51; n = 15) wide. Testis 88–128 (103; n = 11) long, 37–60 (50; n = 11) wide. MCO 62–77 (71; n = 15) long. Germarium 53–75 (63; n = 9) long, 34–54 (40; n = 9) wide.

### Remarks

*Platycephalotrema* Kritsky & Nitta, 2019  was proposed by Kritsky & Nitta [16] for nine dactylogyrids infecting the gill lamellae of flatheads occurring in the Indo-Pacific Ocean: *Platycephalotrema austrinum* Kritsky & Nitta, 2019  from bar-tailed flathead *Platycephalus endrachtensis* Quoy & Gaimard,* Platycephalotrema bassense* (Hughes, 1928) Kritsky & Nitta, 2019  (formerly *Ancyrocephalus bassensis* Hughes, 1928) from southern sand flathead *Platycephalus bassensis* Cuvier; *Platycephalotrema koppa* Kritsky & Nitta, 2019  from dusky flathead *Platycephalus fuscus* Cuvier; *Platycephalotrema macassarense* (Yamaguti, 1963) Kritsky & Nitta, 2019  [formerly *Ancyrocephalus platycephali* Yamaguti, 1953, *Ancyrocephalus macassarensis* Yamaguti, 1963, and *Haliotrema macassarense* (Yamaguti, 1963) Bychowsky & Nagibina, 1971] from bartail flathead *Platycephalus indicus* (Linnaeus); *Platycephalotrema mastix* Kritsky & Nitta, 2019  from *Pl. fuscus* and *Pl. endrachtensis*,* Platycephalotrema ogawai* Kritsky & Nitta, 2019  (type species) from *Platycephalus* sp. 1 & 2 of Nakabo & Kai [20], *Platycephalotrema platycephali* (Yin & Sproston, 1948) Kritsky & Nitta, 2019  [formerly *Haliotrema platycephali* Yin & Sproston, 1948, *Ancyrocephalus platycephali* (Yin & Sproston, 1948) Yamaguti, 1963, and *Pseudohaliotrema platycephali* (Yin & Sproston) Young, 1968] from *Pl. indicus*; *Platycephalotrema sinense* (Yamaguti, 1963) Kritsky & Nitta, 2019 [formerly *Ancyrocephalus thysanophrydis* of Yin & Sproston [32]] from spotted flathead *Cociella punctatus* (Cuvier); *Platycephalotrema thysanophrydis* (Yamaguti, 1937) Kritsky & Nitta, 2019  [formerly *Ancyrocephalus thysanophrydis* Yamaguti, 1937 and *Haliotrema thysanophrydis* (Yamaguti, 1937) Bychowsky & Nagibina, 1971 ] from the Japanese flathead *Inegocia japonica* (Cuvier) and crocodile flathead *Cociella crocodilus* (Cuvier). Kritsky & Nitta [16] also suggested that *Ancyrocephalus vesiculosus* Murray, 1931, *Haliotrema indicum* Tripathi, 1972, *Haliotrema pteroisi* Paperna, 1972, and *Haliotrema swatowense* Yao, Wang, Xia, & Chen, 1998, all parasitic on fishes of the perciform suborder Scorpaenoidei, were possible members of *Platycephalotrema* but did not formally propose the transfers pending further review of each species.

Five dactylogyrid species are known to parasitize the bartail flathead in the Indo-Pacific Ocean: *Platycephalotrema parile* n. sp., *Pt. macassarense*, *Pt. platycephali*, *H. indicum*, and *H. swatowense*. That the bartail flathead serves as host for a comparatively large number of *Platycephalotrema* spp. is not unexpected, however. Puckridge et al. [21], who utilized an analysis of molecular data in development of their hypothesis on the relationships of populations of *Pl. indicus* in the Indo-Pacific Ocean, recognized eight lineages and as a result suggested that *Pl. indicus* may comprised a complex of cryptic species. Whereas dactylogyrids are presumed to have a comparatively high host specificity, specimens parasitizing *Pl. indicus* in the Arabian Gulf, the localities as part of the Western Indian Region [21], could be expected to represent a species of *Platycephalotrema* distinct from those occurring on the bartail flathead in other regions of the host’s natural range. This is borne out by *Pt. macassarense* having been described from the Macassar Strait, Indonesia (Indo Region of [21]) and later reported from the South China Sea off China (NW Pacific Region of [21]) [28, 29, 33], and *Pt. platycephali*, *H. indicum* [herein transferred to *Platycephalotrema* as *Platycephalotrema indicum* (Tripathi, 1959)  n. comb. sp. (see below)], and *H. swatowense* [a junior synonym of *Pt. indicum* (see below)] have been reported from the western Pacific Ocean off China (NW Pacific Region of [21]) [8, 27, 31–33]; *H. indicum* was originally described from the Indo Region of Puckridge et al. [21] by Tripathi [25].

The internal organ systems and the haptoral sclerites of *Platycephalotrema* spp. are morphologically similar and provide few if any features sufficiently distinct to differentiate species within the genus. Species identifications most often depend on comparisons of the respective vaginal sclerites (when present) and the MCOs. Based on the common morphology of their MCOs, *Pt. parile* n. sp. most closely resembles *Pt. ogawai* and the *Platycephalotrema* and *Haliotrema* species infecting the bartail flathead*. Platycephalotrema parile* is differentiated from these species by having a vaginal sclerite with a planate distal flap having thickened margins, which when folded, may form a spine-like structure that extends nearly the entire length of the sclerite (Figs. 15, 16) (a planate flap is absent in *Pt. ogawai*, *H. indicum*, and *H. swatowense*). The vaginal sclerite of *Pt. ogawai* (not figured in the original description of the species by [16]) has two semi-acute lobes and a basal notch (Fig. 17), whereas those of *H. indicum* and *H. swatowense* were reported to have serrated margins (serrated margins absent in *Pt. parile*) (see Fig. 18 in Tripathi [25] and Fig. 2C in Yao et al. [31]). The vaginal sclerite is apparently absent or represented by a “wide cavity” with sclerotized folded walls in *Pt. macassarense* (see [28]), whereas the homologs of *Pt. parile*, *Pt. ogawai*, and *H. indicum* are not cavernous.

Kritsky and Nitta [16], who transferred *Haliotrema platycephali* Yin & Sproston, 1948 to *Platycephalotrema*, suggested that *H. indicum* and *Pt. platycephali* were synonymous but refrained from formally proposing the synonymy pending new collections of the two species from both India and China. Currently, the synonymy of these species remains in doubt. However, the vaginal sclerite of *H. indicum* as depicted by Tripathi [25] and that of *H. swatowense* as shown by Yao et al. [31] are morphologically similar, by having, among other features, serrated or frayed margins. Whereas *H. indicum* and *H. swatowense* are apparently parasitic on the same host species and that they possess similar haptoral, copulatory, and vaginal sclerites, the two nominal species are therefore placed in synonymy, with *H. indicum* having priority. Finally, based on the similarity of the haptoral sclerites as depicted by Tripathi [25] and Yao et al. [31] for *H. indicum* and *H. swatowense*, the presence of a sclerite associated with the vagina in both species, and other features defining *Platycephalotrema* sensu Kritsky & Nitta [16], *H. indicum* is transferred to *Platycephalotrema* as *Platycephalotrema indicum* (Tripathi, 1959) n. comb.
